# Late administration of high-frequency electrical stimulation increases nerve regeneration without aggravating neuropathic pain in a nerve crush injury

**DOI:** 10.1186/s12868-018-0437-9

**Published:** 2018-06-25

**Authors:** Hong-Lin Su, Chien-Yi Chiang, Zong-Han Lu, Fu-Chou Cheng, Chun-Jung Chen, Meei-Ling Sheu, Jason Sheehan, Hung-Chuan Pan

**Affiliations:** 1 Department of Life Sciences, Agriculture Biotechnology Center, National Chung-Hsing University, Taichung, Taiwan; 2 Department of Neurosurgery, Taichung Veterans General Hospital, 1650 Taiwan Boulevard Sec. 4, 40705 Taichung, Taiwan; 3 Department of Medical Research, Taichung Veterans General Hospital, Taichung, Taiwan; 4 Institute of Biomedical Sciences, National Chung-Hsing University, Taichung, Taiwan; 5 Department of Neurosurgery, University of Virginia, Charlottesville, VA USA; 6 Faculty of Medicine, School of Medicine, National Yang-Ming University, Taipei, Taiwan

**Keywords:** Transcutaneous electrical stimulation, Nerve regeneration, Dorsal root ganglion cell, Neuropathic pain

## Abstract

**Background:**

High-frequency transcutaneous neuromuscular electrical nerve stimulation (TENS) is currently used for the administration of electrical current in denervated muscle to alleviate muscle atrophy and enhance motor function; however, the time window (i.e. either immediate or delayed) for achieving benefit is still undetermined. In this study, we conducted an intervention of sciatic nerve crush injury using high-frequency TENS at different time points to assess the effect of motor and sensory functional recovery.

**Results:**

Animals with left sciatic nerve crush injury received TENS treatment starting immediately after injury or 1 week later at a high frequency(100 Hz) or at a low frequency (2 Hz) as a control. In SFI gait analysis, either immediate or late admission of high-frequency electrical stimulation exerted significant improvement compared to either immediate or late administration of low-frequency electrical stimulation. In an assessment of allodynia, immediate high frequency electrical stimulation caused a significantly decreased pain threshold compared to late high-frequency or low-frequency stimulation at immediate or late time points. Immunohistochemistry staining and western blot analysis of S-100 and NF-200 demonstrated that both immediate and late high frequency electrical stimulation showed a similar effect; however the effect was superior to that achieved with low frequency stimulation. Immediate high frequency electrical stimulation resulted in significant expression of TNF-α and synaptophysin in the dorsal root ganglion, somatosensory cortex, and hippocampus compared to late electrical stimulation, and this trend paralleled the observed effect on somatosensory evoked potential. The CatWalk gait analysis also showed that immediate electrical stimulation led to a significantly high regularity index. In primary dorsal root ganglion cells culture, high-frequency electrical stimulation also exerted a significant increase in expression of TNF-α, synaptophysin, and NGF in accordance with the in vivo results.

**Conclusion:**

Immediate or late transcutaneous high-frequency electrical stimulation exhibited the potential to stimulate the motor nerve regeneration. However, immediate electrical stimulation had a predilection to develop neuropathic pain. A delay in TENS initiation appears to be a reasonable approach for nerve repair and provides the appropriate time profile for its clinical application.

**Electronic supplementary material:**

The online version of this article (10.1186/s12868-018-0437-9) contains supplementary material, which is available to authorized users.

## Backgrounds

Any type of nerve repair causes a period of short or long-term change in the connection between the muscle and nerve. The target muscle stayed denervated for several weeks even after immediate repair, leading to denervation-associated atrophy. A more direct method to minimize muscle atrophy is to stimulate the muscle electrically [1, 2]. In general, short-term electrical muscle stimulation after nerve repair potentially reduces muscle atrophy [3, 4]. Although several studies have reported the use of electrical stimulation after immediate nerve repair, the length and stimulation parameter was not adequately determined and these factors remain the subject of debate [5–9].

Neuromuscular electrical stimulation is performed by the application of electrical current directly to the skin surface and underlying muscle to induce a muscle contraction, as well as to retard muscle atrophy during the period of reinnervation [10]. In a prospective, nonrandomized trial, high-tone external muscle stimulation resulted in improvement of tingling, burning, pain, and numbness in diabetic and uremic neuropathy patients [11]. In another study, following external long-term muscle electrical stimulation in uremic neuropathy patients, physical capacity and ulnar motor conduction velocity were markedly improved [12]. To attenuate muscle atrophy and improve function of denervated muscle, stimuli should be applied several times a day at sufficient intensity, pulse duration, and frequency [13].

On the contrary, electrical stimulation can have a detrimental effect in nerve regeneration after crush injury. The transcutaneous electrical stimulation altered the morphology of axon with dark axoplasma, edema, and disorganized cytoarchitecture. In addition, a decrease in axon number was also observed with thinner myelination but with an increased number of Schwann cell nuclei [14]. Electrical stimulation reduced the muscle excitability, neural cell adhesion molecule expression, the integrity of neuromuscular junctions and muscle fiber cross sectional area [6, 8, 13, 15]. Furthermore, a delay of longer than 3 months for stimulation did not increase muscle re-innervation [16]. The stimulation of a partially innervated muscle can also have adverse effects for the remaining nerves because nerve connections to the muscle are formed in an asynchronizing manner, and stimulation at this time compromised functional reinnveration [3].

The timing to start electrical stimulation is very controversial. The significant improvement in twitching tension of crushed nerve was noted only when ES was applied during the middle period (day 12–21) after nerve crush, however, no difference was observed at other time points, suggesting the stimulatory effect occurred only occurred in a specific time window [17]. ES may exert an inhibitory effect on the functional neuromuscular recovery when administrated daily while axons are renewed along the distal nerve stump but before they reach the muscle fibers [6].

Based on a previous review, several controversies exist, including the time profile for initiating neuromuscular stimulation, i.e., immediate, early or delay; electrical frequency; and intensity. In this study, animals with sciatic nerve injury were subjected to the electrical neuromuscular stimulation at the different time profiles and at different stimulation frequencies and intensities to assess the alteration in neurobehavior, electrophysiology, and the associated protein expression. In addition, a primary culture of dorsal root ganglion cells was used to investigate the response by the electrical current.

## Methods

### Nerve crush injury model

Male Sprague–Dawley rats weighing 250–300 g (bought from BioLASCO Taiwan Co.) were used and were anesthetized using isoflurane at 4% in the induction period and 1% in maintenance period. The gluteal splitting method was used to expose left sciatic nerve under the microscope, and the nerve was crushed using a vessel clamp 10 mm from the obturator [18]. The animals were randomly allocated into one of six groups as follows: Group I: sham (n = 6); Group II: nerve crush injury as control (n = 6); Group III (HFI): high-frequency (100 Hz) percutaneous electrical stimulation administrated immediately (n = 12); Group IV (HFL): high-frequency (100 Hz) percutaneous electrical stimulation administrated 7 days after nerve crush (n = 12). Group V (LFI): low-frequency (5 Hz) percutaneous electrical stimulation administrated immediately (n = 6); Group VI (LFL): low-frequency (5 Hz) percutaneous electrical stimulation administrated 7 days after nerve crush (n = 6). The electrical stimulation paradigm featured a treatment consisting of stimulation for 30 min per day for 7 consecutive days using 400 ms of biphasic pulses at 200 μs per phase and 100 or 5 Hz frequency and with 6 s of rest (ElePulsHV-F125, Omron, Japan) [19]. Food and water were provided ad libitum before and after the operation. The animal housing environment was kept under the appropriate condition with 2 animals in a single cage, in a temperature-controlled environment at 20 °C and with alternating light and dark cycles with 12-h intervals. After the experiment, all animals were euthanized using CO2. The care and operation of all animals followed the guidelines recommended by Taichung Veterans General Hospital Institutional Animal Care and Use Committee (IACUC) (Permission No.La-1061455).

### Analysis of motor function recovery

A technician blindly assessed the SFI in the various groups of animals before operation and weekly after the surgery according to our previous report [20, 21]. Several essential parameters were taken from the footprint and all measurements were taken in the experimental and control groups. An SFI of 0 indicated normal function and − 100 represented total loss of motor function.

### Nociceptive behaviors

Mechanical allodynia was tested blindly by using von Frey hairs (Touch-Test Sensory Evaluator, North Coast Medical, Inc), as previously described by our group [20, 21]. Von Frey hairs were applied in a series of grams to touch the hind paw bilaterally five times for 5-s intervals when the hind paw was placed appropriately on the platform. The withdrawal threshold was considered to be the force (gram) of the hair that caused hind-paw withdrawal in at least four out of the five applications. Thermal hyperalgesia was evaluated via a hot-plate test (Technical& Scientific Equipment GmbH, TSE systems) according to the pervious procedure [21]. The withdrawal latency was recorded as the interval of the time from which the rat touched the 52 °C hotplate to the time of withdrawal of the paw. A maximal cut-off of twenty-seconds was used to prevent paw tissue injury.

### Catwalk gait analysis

The CatWalk XT gait analysis has been previously described by our group [21]. Quantitative analysis of the data included the following parameters: step sequence distribution, regularity index (RI), print area, duration of swing and stance phases, and intensity. The data were presented as the ratio of the measurement for the left side divided by that for the right side.

### Evoked potential of the somatosensory cortex

Evoked potential measurements have been previously published by our group [20, 21]. In brief, one active electrode was threaded into the dural surface of the somatosensory area (3 mm lateral and 2 mm posterior to the bregma). Another electrode was placed as a reference over the maxillary area at approximately 20 mm from the active electrode. A stimulation intensity of 20 mA with 20–2000 Hz filtration was applied over the sciatic nerve 1 cm proximal to the injury area. The data for conduction latency and evoked potential were presented as the ratio of the measurement for the right side to the left side, to minimize the effects of anesthesia.

### Isolation and cultured dorsal root ganglion cells (DRGs) subjected to electrical stimulation

Dorsal root ganglia cells were dissected from embryonic Sprague–Dawley rat at the embryonic days 14–15 according to previous report [20, 22, 23]. The DRGs were incubated with 0.25% trypsin at 37 °C for 15 min and were dissociated, washed and re-suspended with Neurobasal medium containing 2% B27 (sigma, Inc.), 0.3% l-glutamine and 100 ng/ml nerve growth factor. Finally, these cells were cultured in the dish at a density of 1 × 10^4^ cells per ml of medium (16-well array station, ECIS model 800). Next, the cells were maintained and cultured in an incubator at 37 °C and 5% CO_2_. The cells were recognized by neuronal markers (βIII tubulin) before the experimental process and were subjected to electrical stimulation for duration of 30 min at frequencies of 5 and 100 Hz with 50 mA.

### Western blot analysis

The distal end of the nerve, muscles, dorsal root ganglion cells, and brain (hippocampus/cortex) were harvested 4 weeks after the various treatment and proteins were extracted. The cell lysate of dorsal root ganglion cells after electrical stimulation were collected to determine the expression of synaptophysin, TNF-α, and NGF. Proteins (50 μg) were resolved by SDS–polyacrylamide gel electrophoresis and were transferred onto a blotting membrane [20]. After blocking with non-fat milk, the membranes were incubated with antibodies against S-100 (Neomarkers, 1:500 dilution), NF (Cellsignal, 1:1000 dilution), synaptophysin (Abcam, 1:500 dilution), TNF-α (Abcam, 1:1000 dilution), and NGF-R (1:1000, Abbiotec) overnight at 4 °C. The intensity of the protein bands was determined by a computer image analysis system (IS1000, Alpha Innotech Corporation, CA, USA).

### Immunohistochemistry staining

Dorsal root ganglion cell culture after electrical stimulation and serial 8-mm-thick section of nerve, muscle, dorsal root ganglion cells, and the brain were cut using a cryostat, and mounted on superfrost/plus slides (Menzel-Glaser, Braunschweig, Germany) were subjected to immunohistochemistry using antibodies against NGF-R (1:1000, Abbiotec), S-100(1:200, Serotec), neurofilament(1:200, Millipore), anti-synaptophysin (Abcam, 1:200 dilution), and anti-TNF-α (Abcam, 1:300 dilution) to detect the inflammatory response associated with nerve regeneration in sciatic nerve, dorsal root ganglion cells, and the brain. The immunoreactive signals were observed using AF 488 donkey anti–mouse IgG and AF594 donkey anti-rabbit (Invitrogen; 1:200 dilutions) and were then viewed using an Olympus BX40 Research Microscope.

### Statistical analysis

Data are expressed as the mean ± SE (standard error). The SFI and CatWalk data were analyzed via repeated-measure ANOVA followed by Bonferroni’s multiple comparison method. The statistical significance of the differences among the groups was determined via one–way analysis of variance (ANOVA) followed by Dunnett’s test. A *p* value < 0.05 was considered significant.

## Results

### Immediate high-frequency electrical stimulation caused significant motor function improvement but cause a predisposition to neuropathic pain

**Fig. 1:**
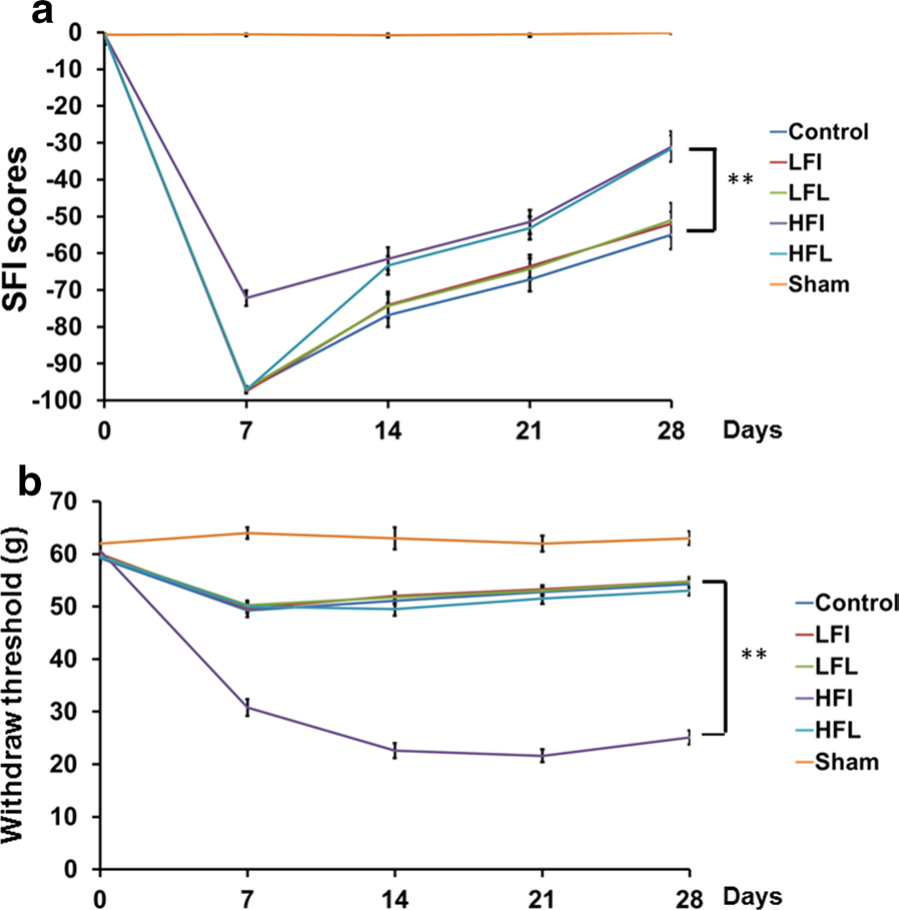
Illustration of SFI and mechanical withdrawal threshold in different treatment groups at different time profiles. **a** Representative of SFI scores related to different time frames subjected to different treatments. **b** Representative of mechanical withdrawal threshold after different treatments given at different time points. Sham; Crush; HFI; HFL; LFI; LFL: see text. **p < 0.01, n = 6

These animals were subjected to different treatments evaluated by SFI (motor function) and mechanical withdrawal threshold (sensory function), as illustrated in Fig. 1. SFI analysis demonstrated no significant improvement after low-frequency electrical stimulation with either immediate or late treatment compared to that in the control group. High-frequency stimulation, immediate electrical stimulation exerted a significant improvement as early as at day 7 with a steeper slope compared to that in the other groups. However, late electrical stimulation delayed improvement in the beginning but reached the effects observed with immediate high-frequency treatment at day 14. Overall, only high-frequency stimulation-either immediate or late-showed the significant improvement in motor function compared to the control or low-frequency electrical stimulation (Fig. 1a).

In the mechanical withdrawal threshold assessment, a significant decrease in mechanical withdrawal was observed after immediate high-frequency treatment compared to that observed in the other groups. There were no significant differences in mechanical withdrawal among the control, HFL, LFI, and LFL groups (Fig. 1b). These observations suggest that immediate high-frequency electrical stimulation exerted a significant enhancement of motor function from the early period that lasted to the final point of assessment; however, this stimulation carried a higher risk of neuropathic pain. The late high-frequency electrical stimulation showed delayed improvement of motor function compared to that in the HFI group but approached the final outcome of the HFI group, without the development of neuropathic pain.

Based on the above assumption and in accordance with the guidelines of NC3Rs Animal Research: Reporting In Vivo Experiments (ARRIVE), to reduce the numbers of animals used in experimental studies, the remaining part of the study focused on determining the appropriate time profile to initiate the TENS treatment of high-frequency electrical stimulation through either immediate or late administration (Additional file 1).

### High-frequency electrical stimulation increased nerve myelination either immediate or late administration

**Fig. 2:**
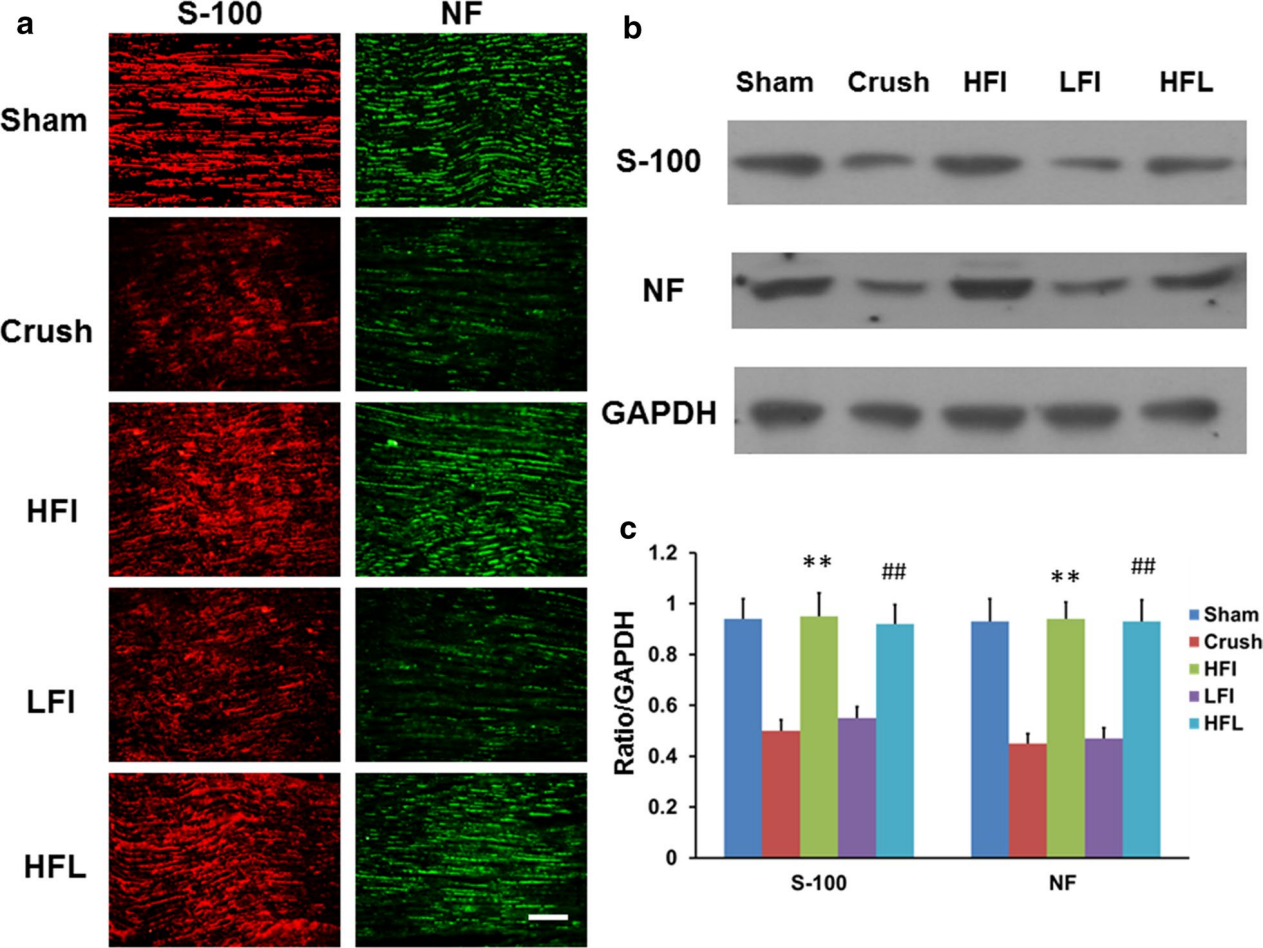
Expression of myelination markers following electrical stimulation 1 month after injury. **a** Immunohistochemistry staining of S-100 and neurofilament in different treatment groups. **b** Representative of western blot analysis in different treatment groups. **c** Quantitative assessment of western blot analysis. N = 3; Bar length = 100 μm; **p < 0.01; Sham, HFI, HFL, LFI: see text; NF = neurofilament

For further confirmation of the nerve regeneration potential subjected to immediate or late high-frequency electrical stimulation, the sciatic nerve was harvested one month after injury. Theses nerves were subjected to immunohistochemistry analysis of S-100 and neurofilament (Fig. 2a, b). There was a significantly higher expression of myelination markers such as S-100 and neurofilament for immediate and late high-frequency electrical stimulation compared to that for the control and immediate low-frequency electrical stimulation (Fig. 2c). This result suggested that high-frequency electrical stimulation through either immediate or late administration exhibited potential for nerve regeneration.

### Immediate high-frequency electrical stimulation caused higher expression of the inflammatory response in the dorsal root ganglion and brain

**Fig. 3:**
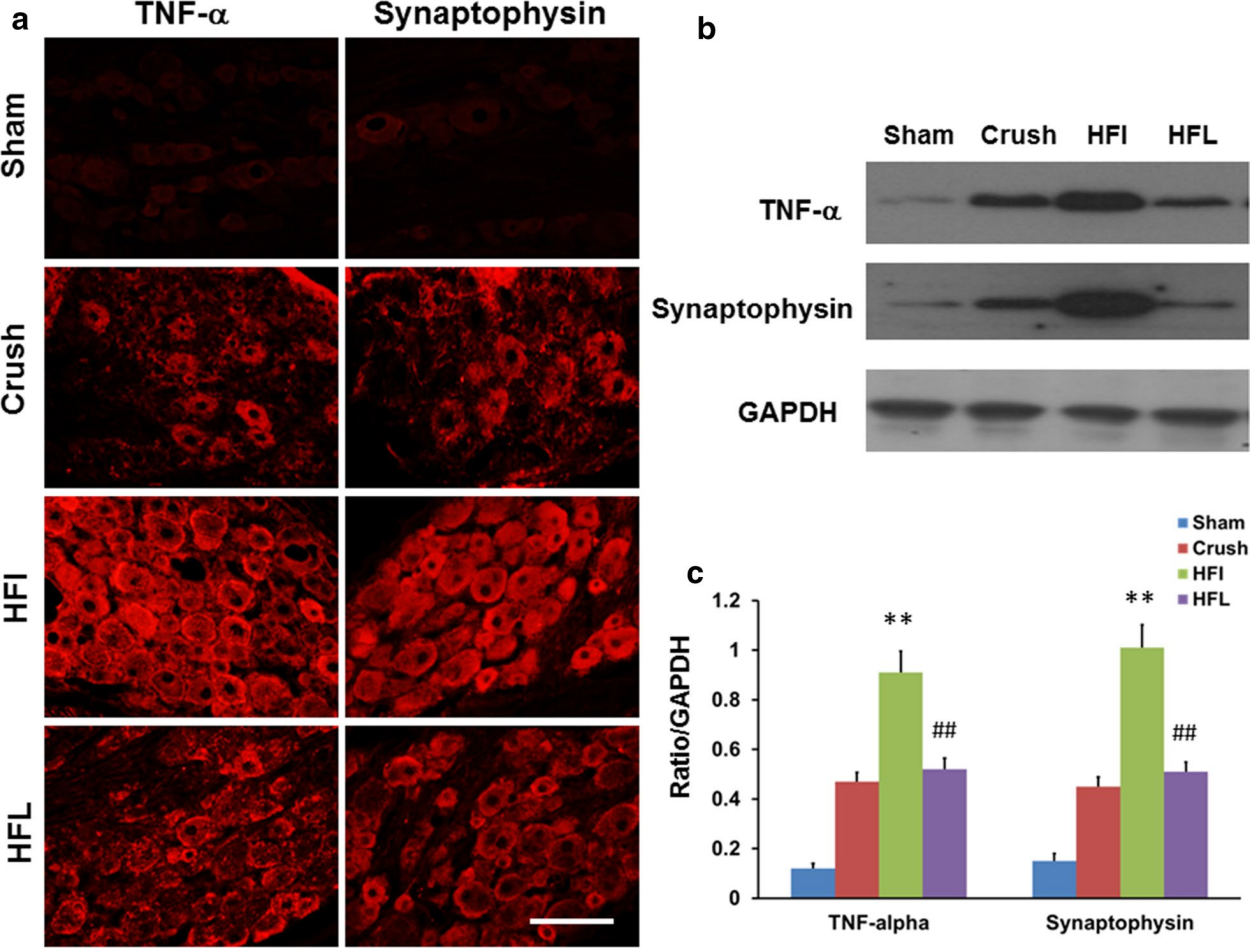
Expression of synaptophysin and TNF-α in dorsal root ganglion cells subjected to high-frequency electrical stimulation. **a** Representative of immunohistochemical staining of synaptophysin and TNF-α in dorsal root ganglion cells under different treatments. **b** Representative of western blot of synaptophysin and TNF-α in dorsal root ganglion tissue in the different treatment groups. **c** Quantitative analysis of western blot of synaptophysin and TNF-α in different treatment groups. Bar length = 100 μm; Sham, Crush, HFI, HFL: see text; n = 3; **p < 0.01 indicated a significant difference relative to the crush group; ##*p* < 0.01 indicated a significant difference relative to HFI

**Fig. 4:**
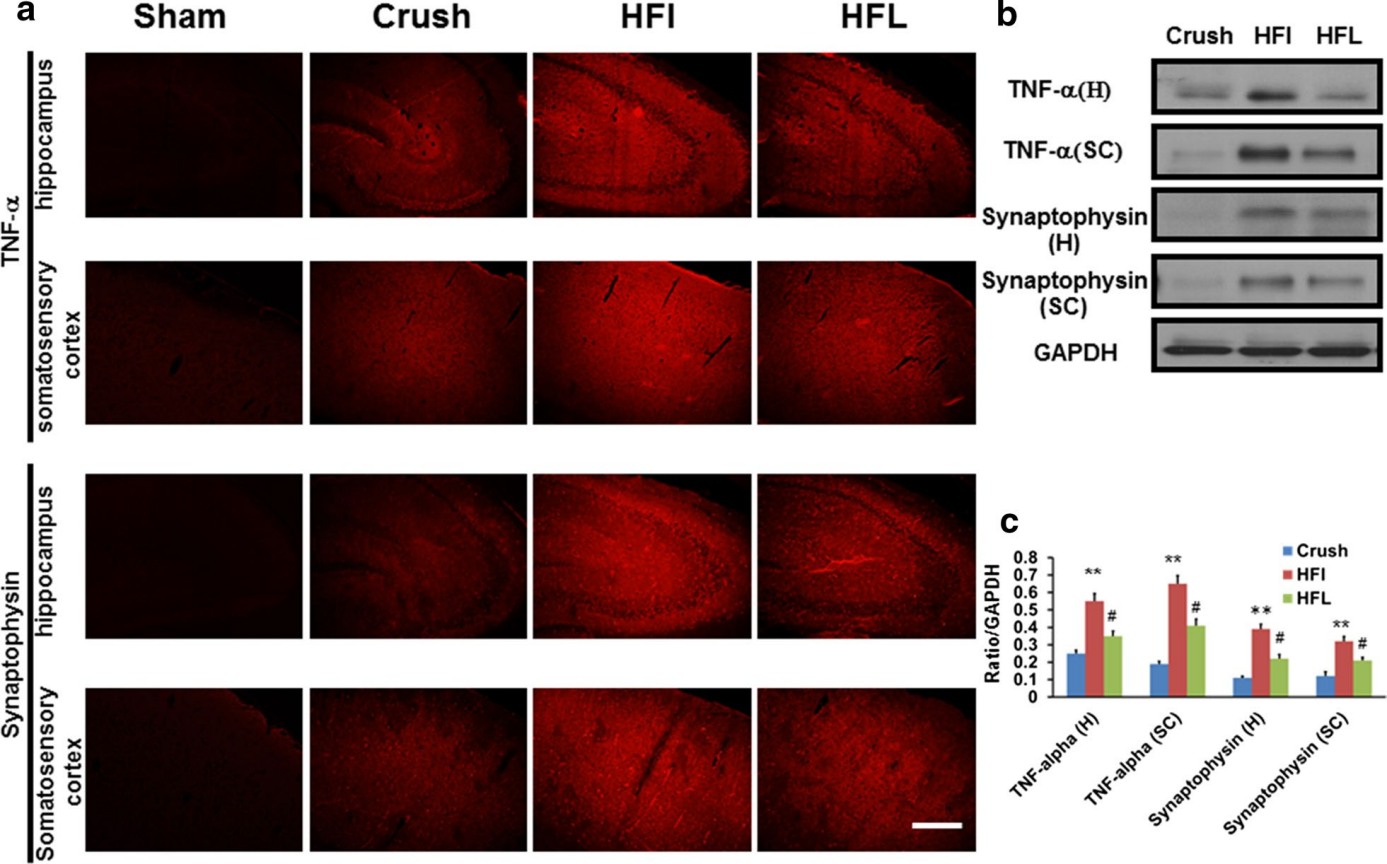
Expression of synaptophysin and TNF-α in the brain after high-frequency electrical stimulation one month after injury. **a** Representative of synaptophysin and TNF-α in the hippocampus and somatosensory cortex in different treatment groups **b** Representative of western blot analysis of synaptophysin and TNF-α in the different treatment groups. **c** Quantitative analysis of western blot of synaptophysin and TNF-α for different treatment groups. Sham, Crush, HFI, HFL: see text; N = 3; **p < 0.01 indicated the significant difference compared to crush group; #*p* < 0.05 indicated the significant difference compared to HFI; Bar length = 100 μm

Synaptophysin and TNF-α expression in the dorsal root ganglion and the somatosensory cortex and hippocampus represents the severity of neuropathic pain. Immediate high-frequency electrical stimulation resulted in a significantly higher expression of synaptophysin and TNF-α in the dorsal root ganglion compared to the control treatment or high-frequency late electrical stimulation (Fig. 3a–c). Higher expression of synaptophysin and TNF-α in the somatosensory cortex and hippocampus was also noted in the immediate group compared to that in the late high-frequency and control groups (Fig. 4a–c).

### Alteration of CatWalk gait analysis and increased evoked potential in the somatosensory cortex after immediate high-frequency electrical stimulation

**Fig. 5:**
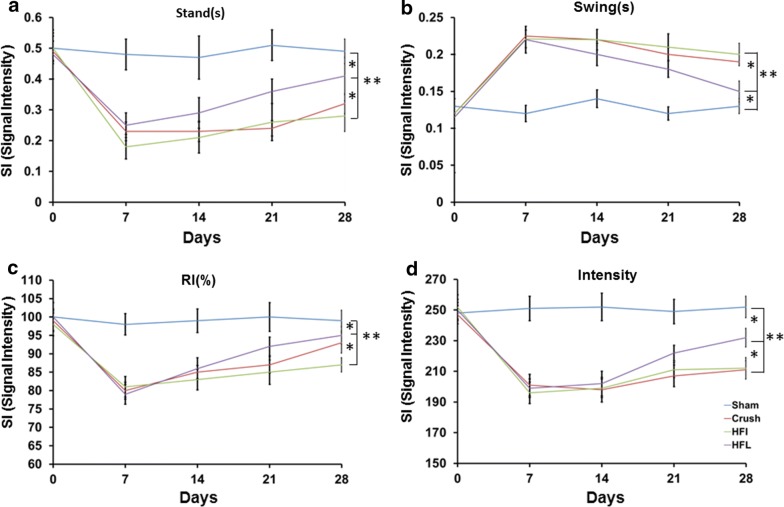
CatWalk gait analysis after high-frequency electrical stimulation. **a** Representative of stands in different time frame subjected to different treatments. **b** Representative of swings in different time frames under different treatments. **c** Representative of RI in different time frames under different treatments. **d** Representative of hind paw intensity in different time frames under different treatments. Sham, Crush, HFI, HFL: see text; N = 6; **p < 0.01 indicated a significant difference related to the crush group

The Catwalk gait analysis was used to examine the motor and sensory functions. Increased intensity, decreased stance, increased swing, and decreased regularity are indicative of motor function improvement. However, increased neuropathic pain produces the opposite trend. Late high-frequency electrical stimulation produces significant improvements in intensity, stance, swing, and regularity index compared to immediate high-frequency electrical stimulation or the control groups (Fig. 5a–d). This phenomenon was because the effect of immediate high-frequency electrical stimulation in motor function was compromised by the increased sensory functional impairment.

**Fig. 6:**
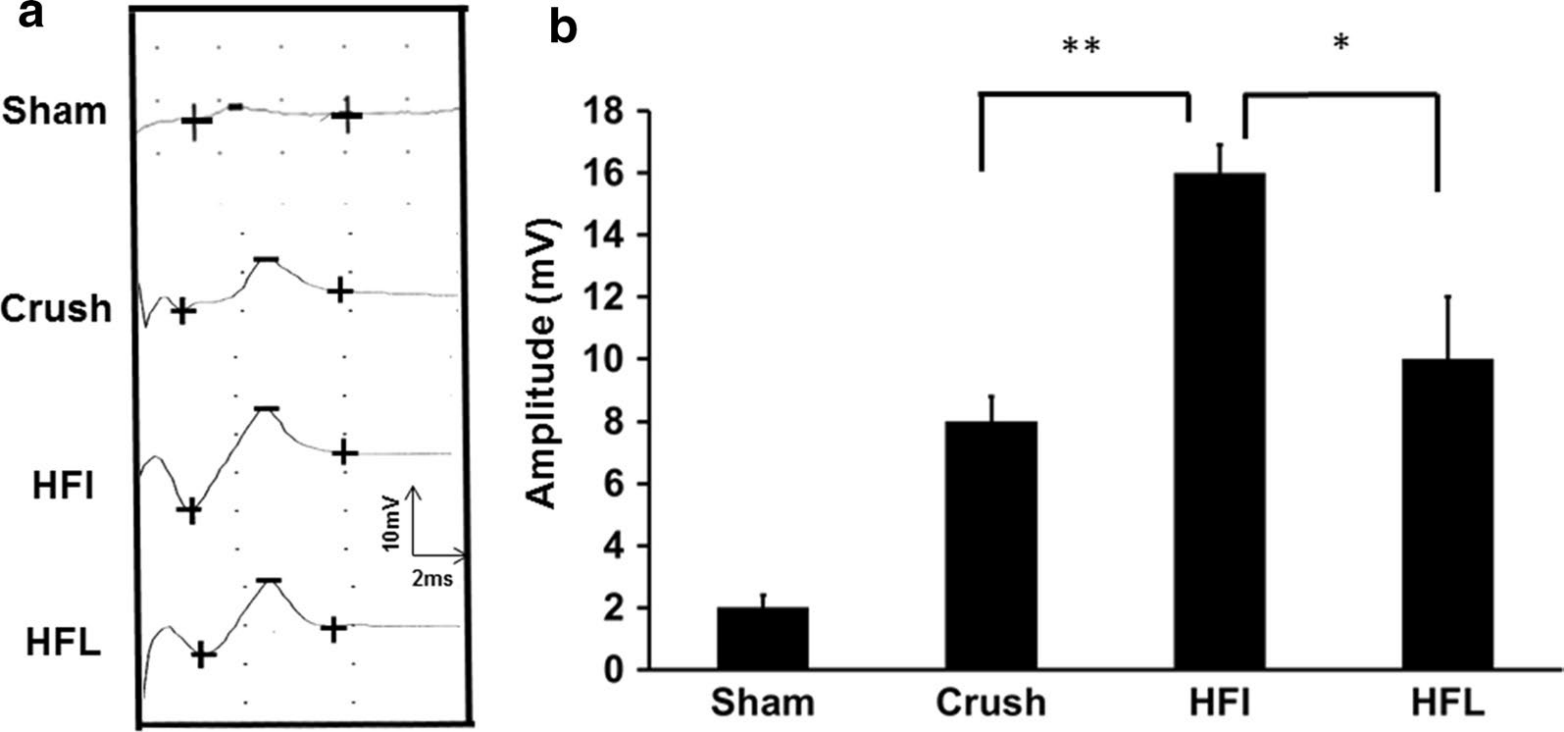
Somatosensory evoked potential after high-frequency electrical stimulation. **a** Representative of somatosensory evoked potential in different treatment groups. **b** Quantitative analysis of somatosensory evoked potential in different treatment groups. Sham, Crush, HFI, HFL: see text; n = 3; *p < 0.01, **p < 0.01

Increased evoked potential in the central nervous system is positively correlated with a peripheral nervous system injury. The increased evoked potential amplitudes in the somatosensory cortex were significantly higher after immediate high-frequency electrical stimulation than after late high-frequency electrical stimulation or the control (Fig. 6a, b). The somatosensory evoked potential data further confirmed the alteration in the neurobehavioral and histomorphological characteristics.

### Electrical stimulation induced the expression of the inflammatory response in dorsal root ganglion cell culture

**Fig. 7:**
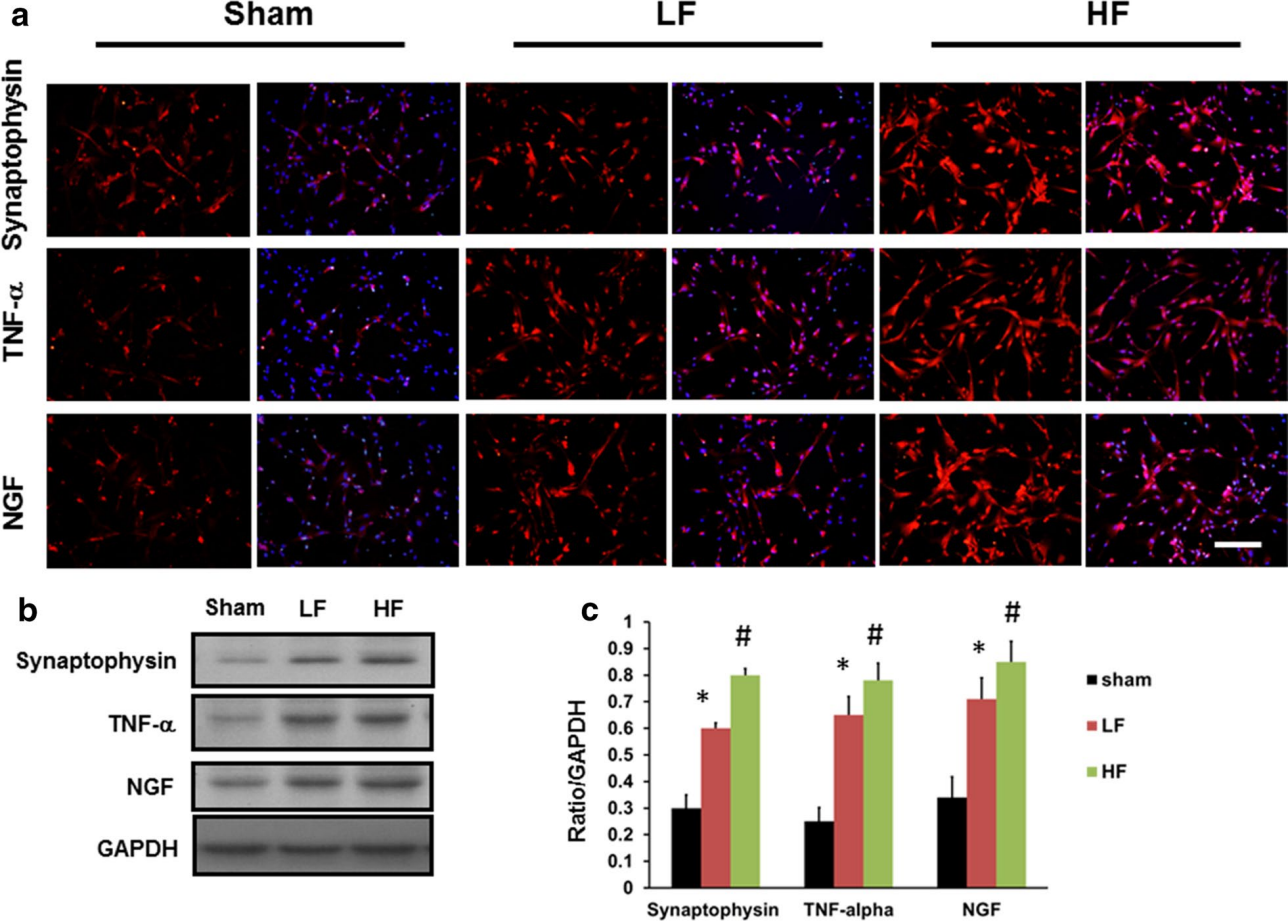
Expression of synaptophysin, TNF-α and NGF in dorsal root ganglion cell culture subjected to electrical stimulation **a** Representative of immunohistochemical staining of synaptophysin, TNF-α and NGF over the dorsal root ganglion cell culture subjected to different frequencies of electrical stimulations. **b** Representative of western blot analysis of synaptophysin, TNF-α and NGF over the dorsal root ganglion cell culture subjected to different frequency electrical stimulations. **c** Quantitative analysis of the western blot of synaptophysin, TNF-α and NGF in the dorsal root ganglion cell culture subjected to different frequencies of electrical stimulation. Sham: dorsal root ganglion cell culture without electrical stimulation; LF: low-frequency electrical stimulation; HF: high-frequency electrical stimulation; *p < 0.01 indicated a significant difference compared to the sham group; #*p* < 0.05 indicated a significant difference compared to HF; bar length = 100 μm; n = 3

To assess the inflammatory response in dorsal root ganglion cells stimulated by direct electrical stimulation, dorsal root ganglion cells were harvested and subjected to electrical stimulation. Immunohistochemistry staining demonstrated that there was increased expression of synaptophysin, TNF-α and NGF-R in the dorsal root ganglion cell culture, which was related to the difference in intensity of the electrical stimulation frequency (Fig. 7a, b). Quantitative analysis demonstrated significantly higher expression of synaptophysin, TNF-α and NGF-R after high-frequency electrical stimulation compared to that after low-frequency electrical stimulation and the sham (Fig. 7c). Hence, high-frequency electrical stimulation harbored the potential to stimulate the dorsal root ganglion cells to express the inflammation associated proteins.

## Discussion

High-frequency electrical stimulation harbored the potential to better augment nerve regeneration compared to low-frequency electrical stimulation; however, their regeneration ability was compromised by their direct electrical effect on sensory function impairment. Thus, the appropriate time profile to start the treatment after nerve injury is unclear. In this study, we found that high-frequency electrical stimulation more effectively primed dorsal root ganglion cells to express inflammatory cytokines such as TNF-α, synaptophysin, and NGF. In animal studies, significantly increased nerve regeneration was noted after high-frequency electrical stimulation administrated either immediately or late; however, immediate electrical stimulation exhibited a higher potential to lead to neuropathic pain. A delay in high-frequency electrical stimulation appeared to be an appropriate time to initiate the electrical stimulation after nerve injury.

Electrical stimulation plays an important role in the treatment of neuromuscular junction disease. There are many method and types of electrical stimulation. The most common method of transcutaneous electrical stimulation used an electrical current of 90-130 Hz [24]. In another study, immediate high-frequency (100 Hz) electrical stimulation of the muscle exerted significantly a significant increase in the expression of neurotrophic factors, which contributed to neurological improvement [25]. In a diabetes animal study, high-frequency electrical stimulation (200 Hz) exerted a greater myelination effect than low-frequency stimulation (20 Hz) [26]. In contrast, a low-frequency percutaneous electrical stimulation of 2 Hz enhanced the mean value of axonal density, blood vessel number and axon outgrowth through the nerve graft [27, 28]. In our study, we found that high-frequency percutaneous electrical stimulation better improved the neurological outcome including neurobehavior and maturation of myelinization compared to low-frequency electrical stimulation. The results paralleled previous studies that demonstrated that high-frequency electrical stimulation contributing to better nerve regeneration potential than low-frequency electrical stimulation.

Percutaneous electrical stimulation was shown to alleviate pain in cases of musculoskeletal pain, arthritis pain, low back pain, neuropathic pain, and post-operative pain [22, 23, 29, 30]. Low-frequency electrical stimulation induced analgesia by inhibiting pain transmission through the recruitment of the descending inhibitory system; however, high-frequency (80–100 Hz) stimulation activated the gate control by stimulation A-beta fibers [31].There were various inconsistencies amongst previous studies with respect to the therapeutic effect according to the frequencies tested [32]. In our study, high-frequency electrical muscle stimulation only improved the nerve regeneration without producing an analgesic effect. Furthermore, there was no pain relief following immediate electrical stimulation, however the stimulation predisposed the sensory system to inflammatory changes. Considering the nerve regeneration potential of high-frequency electrical stimulation, a delay in the initiation of stimulation appears advisable.

Dorsal root ganglion cells are located in the intervertebral foramen of the spinal cord and involve the sensory neuron; these cells respond to peripheral nerve injury. The increased expression of TNF-α, IL1β, and synaptophysin reflected the intensity of the neuropathic pain. The attenuation of these inflammatory cytokines paralleled the decreased neuropathic pain response [20, 21]. In vitro analysis demonstrated that high-frequency electrical stimulation exerted a greater potential for the dorsal root ganglion cells to express an inflammatory response compared to the low-frequency electrical stimulation. In the vivo analysis, immediate high frequency electrical stimulation resulted in a significantly higher expression of inflammatory cytokines in the dorsal root ganglion compared to late high-frequency electrical stimulation and low-frequency electrical stimulation either at the immediate or late time profiles. This result confirmed that a delay in high-frequency electrical stimulation is appropriate to facilitate nerve regeneration without increasing the risk of neuropathic pain.

Electrophysiological and biochemical alterations have been well documented to occur in the somatosensory system of the brain after peripheral nervous system injury. An increased expression of TNF-α and synaptophysin in the somatosensory cortex and hippocampus indicate a stronger response to peripheral nerve injury, and an attenuation of this response was noted during a decrease in neuropathic pain [20]. In addition, the evoked potential in the somatosensory cortex was in line with the severity of neuropathic pain with respect to both behavioral and biochemical aspects. This study showed a significantly high amplitude of the evoked potential following immediate high-frequency electrical stimulation. Both electrophysiological and molecular biology data confirmed that immediate high-frequency electrical stimulation had a potential to induce neuropathic pain.

The CatWalk gait XT system is a comprehensive and sensitive tool for the determination of gait alteration in motor and sensory function impairment at the same time profile compared to using the SFI and allodynia analyses alone [20, 33]. Motor function improvement is reflected by an increased paw intensity, increased stance, decreased swing and a decreased regularity index. However, sensory impairment is indicated by reciprocal trends [33]. Immediate high-frequency electrical stimulation showed a significant improvement in SFI but decreased the pain threshold in the mechanical allodynia test. The Catwalk gait analysis demonstrated a increased paw density, increased stance, decreased swing and reduced regularity index in motor function accompanying the sensory impairment. In the CatWalk gait analysis, we found that the increased regularity index and decreased paw intensity following immediate high-frequency electrical stimulation was due to the compromised effect of sensory and motor function alterations.

There were some limitations in this study. First, the nerve crush model cannot present the typical sensory impairment observed in well-established neuropathic models such as chronic nerve constriction injury, tibia nerve resection, or nerve root ligation model. However, we aimed to report motor function improvement without aggravation of sensory function through the investigation of different treatment time profiles and the administration of high or low electrical frequency. The nerve-crush injury model consisted of motor and sensory function impairment and was the appropriate model in this study. Second, dorsal root ganglion cell culture, subjected to either high or low-frequency electrical stimulation, did not reflect the status of late TENS biology. However, we intend to investigate the ability of high- and low-frequency electrical stimulation to prime dorsal root ganglion cells to initiate the inflammatory response to increase the nerve regeneration and improve animal behavior after high-frequency electrical stimulation but mitigate the risk of impaired sensory function.

## Conclusion

Both immediate and late administration of high-frequency electrical stimulation exerted a significantly greater motor function improvement than low-frequency electrical stimulation at comparable time points. However, immediate high-frequency electrical stimulation caused a significantly lower pain threshold than late high-frequency or low-frequency stimulation delivered at the immediate or late time points. There, a delay in the initiation of TENS appears to afford a reasonable treatment approach after nerve repair and provides an appropriate time frame for clinical administration.

## Additional file

**Additional file 1**. The ARRIVE guidelines checklist.

## Abbreviations

TENS: transcutaneous neuromuscular electrical nerve stimulation
NF: neurofilament
SFI: sciatic function index
CMAP: compound muscle action potential
DRGs: dorsal root ganglion cells
