# Supplementary material for: Late administration of high-frequency electrical stimulation increases nerve regeneration without aggravating neuropathic pain in a nerve crush injury
Source: BMC Neurosci. 2018 Jun 25;19:37. doi: 10.1186/s12868-018-0437-9 (PMC6020201; doi:10.1186/s12868-018-0437-9)
Supplement: Supplementary file 1 — Additional file 1. The ARRIVE guidelines checklist. [file 12868_2018_437_MOESM1_ESM.pdf]

The ARRIVE Guidelines Checklist

	ITEM	RECOMMENDATION	Section/ Paragraph
Title	1	Late administration of high frequency electrical stimulation increases nerve regeneration without aggravating neuropathic pain in a nerve crush injury	Page 1
Abstract	2	Sprauge-Dawely rats weighting 250 to 300gm were used in this study. The operative procedure was conducted by using left gluteal-splitting method by a vessel clamp to crush left sciatic nerve 1cm above the trifurcation. After operation, the animals were allowed to receive TENS treatment starting immediately, or one week later intervened by high (100Hz) and low frequency (2Hz) as a control. These animals were assessed by neurobehavioral, electrophysiology and immunohistochemistry study for assessment of nerve regeneration and neuropathic pain. In addition, primary cultures of dorsal root ganglion cells were used to investigate the inflammatory response by the electrical current.	Page 3-4
INTRODUCTION			
Background	3	High frequency transcutaneous neuromuscular electrical nerve stimulation (TENS) is currently used for administration of electrical current in denervated muscle to alleviate muscle atrophy and enhance motor function, but the time window for achieving benefit (i.e. either immediate or delayed) is still undetermined.	Page 5-7
Object	4	In this study, we conducted sciatic nerve crush injury intervened by high frequency TENS at different time points to assess the effect of motor and sensory functional recovery.	Page 7
METHODS			
Ethic statement	5	Sprague-Dawley rats weighing 250-300 g were used in this study. All animals of care and operation were under the guidelines recommended by Taichung Veterans General Hospital Institutional Animal Care and Use Committee (IACUC) (Permission No.La-1061455).	Page 8
Study deisgn	6	After the nerve crush, the animals were randomly allocated into one of five groups as follows: Group I: nerve crush a control (n=6); Group II (HFI): high frequency (100Hz) percutaneous electrical stimulation administrated immediately (n=12); Group III (HFL): high frequency (100Hz) percutaneous electrical stimulation administrated 7 days after nerve crush (n=12). Group IV (LFI): Low frequency (5Hz) percutaneous electrical stimulation administrated immediately (n=6); Group V (LFL): low frequency (5Hz) percutaneous electrical stimulation administrated 7 days after nerve crush (n=6).	Page 8
Experimental Procedure	7	Forty-Two Sprague-Dawley rats weighing 250-300g (bought from BioLASCO Taiwan Co.) was used with anesthetization of 4% isoflurane in induction and 1% in maintenance period. The gluteal splitting method was used to expose left sciatic nerve under the microscope and crushed by a vessel clamp 10mm from the obturator [18]. The immediate recovery of animals was	Page 8-13

		monitored closely in the recovery cage with room temperature of 24-25 ℃ until they are able to maintain the sternal recumbency. For the decrease of post- operative pain, these animals received intramuscular injection of ketoprofen 5mg/kg q12 hours for one day. After the nerve crush, the animals were randomly allocated into one of five groups as follows: Group I: nerve crush a control (n=6); Group II (HFI): high frequency (100Hz) percutaneous electrical stimulation administrated immediately (n=12); Group III (HFL): high frequency (100Hz) percutaneous electrical stimulation administrated 7 days after nerve crush (n=12). Group IV (LFI): Low frequency (5Hz) percutaneous electrical stimulation administrated immediately (n=6); Group V (LFL): low frequency (5Hz) percutaneous electrical stimulation administrated 7 days after nerve crush (n=6). The wound was closely observed and evaluated every day and the stitches were removed 10 days after operation. In the electrical stimulation, the paradigm featured a treatment consisting of 30 minutes per day for 7 consecutive days using 400ms of 100 or 5 Hz frequency and 200 μs per phase biphasic pulses with 6 seconds of rest (ElePulsHV-F125, Omron, Japan) [19]. The rehabilitation program was conducted on a metal mesh every week. Food and water were provided ad libitum before and after the operation. The animal housing environment was kept in the appropriate condition with 2 animals in a single cage, in a temperature-controlled environment at 20 ℃ and alternating light and dark cycles with 12 hour intervals. After the experiment, all animals were euthanized with CO_2. All animals of care and operation were under the guidelines recommended by Taichung Veterans General Hospital Institutional Animal Care and Use Committee (IACUC) (Permission No.La-1061455). The animals received motor and sensory function assessment (SFI, nociceptive behaviors, Catwalk gait analysis) pre-operative and weekly after operation till the end of experiment and then subjected for immunohistochemistry staining and electrophysiology (evoked potential, CMAP, conduction latency) 4 weeks after operation (n=6 for each group) (total of 30 animals). At the end of experiment, brain, dorsal root ganglion, and nerve of these animals in were also used for western blot analysis in group II and III (total of 12 animals).	
Experimental Animals	8	Male Sprague-Dawley rats weighing 250-300 g were bought from BioLASCO Taiwan Co., Ltd and used in this study.	Page 8
Housing and husbandry	9	The animal housing environment was kept in the appropriate condition with 2 animals in a single cage, in a temperature-controlled environment at 20 ℃ and alternating light and dark cycles with 12 hour intervals.	Page 8
Sample size	10	The animals received motor and sensory function assessment (SFI, nociceptive behaviors, Catwalk gait analysis) pre-operative and weekly after operation till the end of experiment and then subjected for immunohistochemistry staining and electrophysiology (evoked potential, CMAP, conduction latency) 4 weeks after operation (n=6 for each group) (total of 30 animals). At the end of experiment, brain, dorsal root ganglion, and nerve of these animals in were also used for western blot analysis in group II and III (total of 12 animals).	Page 8

Allocating animals To experimental group	11 After the nerve crush, the animals were randomly allocated into one of five groups as follows: Group I: nerve crush a control (n=6); Group II (HFI): high frequency (100Hz) percutaneous electrical stimulation administrated immediately (n=12); Group III (HFL): high frequency (100Hz) percutaneous electrical stimulation administrated 7 days after nerve crush (n=12). Group IV (LFI): Low frequency (5Hz) percutaneous electrical stimulation administrated immediately (n=6); Group V (LFL): low frequency (5Hz) percutaneous electrical stimulation administrated 7 days after nerve crush (n=6).	Page 8
Experimental Outcome	12 These animals were assessed by neurobehavioral, electrophysiology and immunohistochemistry study for assessment of nerve regeneration and neuropathic pain. In addition, primary cultures of dorsal root ganglion cells were used to investigate the inflammatory response by the electrical current.	Page 13-18
Statistical Method	13 Data are expressed as mean ± SE (standard error). The results of SFI and Catwalk data were analyzed by repeated-measurement of ANOVA followed by Bonferroni's multiple comparison method. The statistical significance of differences between groups was determined by one–way analysis of variance (ANOVA) followed by Dunnett's test. A p value less than 0.05 was considered significant.	Page 13
RESULTS		
Baseline Data	14 In SFI gait analysis, high frequency electrical stimulation either immediate or late admission exerted significant improvement as compared to low frequency electrical stimulation either immediate or late administration. In allodynia assessment, immediate high frequency electrical stimulation caused significantly decreased pain threshold as compared to late high frequency or low frequency at immediate or late time points. In immunohistochemistry staining or western blot of S-100 and NF-200 either immediate or late high frequency electrical stimulation showed a similar effect but superior to those achieved with low frequency stimulation. Immediate high frequency electrical stimulation showed significant expression of TNF-alpha and synaptophysin over the dorsal root ganglion, somatosensory cortex, and hippocampus as compared to late electrical stimulation and this trend paralleled the result of somatosensory evoked potential. The Catwalk gait analysis also showed that immediate electrical stimulation led to a significantly high regularity index. In primary dorsal root ganglion cells culture, high frequency electrical stimulation also exerted significantly high expression of TNF-alpha, synaptophysin, and NGF in accordance with those in-vivo results.	Page 13-18
Number	15 The animals received motor and sensory function assessment (SFI, nociceptive behaviors, Catwalk gait analysis) pre-operative and weekly after operation till the end of experiment and then subjected for immunohistochemistry staining and electrophysiology (evoked potential, CMAP, conduction latency) 4 weeks after operation (n=6 for each group) (total of 30 animals). At the end of experiment, brain, dorsal root ganglion, and nerve of these animals in were also used for western blot analysis in group II and III (total of 12	Page 13-18

		animals).	
Outcome<br>And estimation	16	These animals were subjected to different treatment profile evaluated by SFI (motor function) and mechanic withdraw threshold (sensory function) illustrated in Figure 1. In SFI analysis, there were no significant improvement in low frequency electrical stimulation with either immediate or late treatment as compared to control group. In high frequency stimulation, immediate electrical stimulation exerted significant improvement as early as at day of 7 with a steeper slope as compared to the other groups. However, late electrical stimulation exerted a delay of improvement in the beginning but reached the results of immediate high frequency treatment at day 14. On the whole, only the high frequency-- either immediate or late-- showed the significant improvement of motor function as compared to control or low frequency electrical stimulation (Figure 1A).<br><br>In the mechanical withdrawn threshold assessment, there were only a significant decrease of mechanic withdraw in the immediate high frequency treatment as compared to the other groups. There were no significant difference of mechanic withdraw among the groups of control, HFL, LFI, and LFL (Figure 1 B). This suggest high frequency immediate electrical stimulation exerted a significant enhancement of motor function from the early period and lasted to the final point of assessment, but it carried a higher risk of neuropathic pain. The late high frequency electrical stimulation showed delayed improvement of motor function compared to HFI group and approached the final outcome of HFL group, but without the development of neuropathic pain.<br><br>For further confirmation of the nerve regeneration potential subjected to immediate or late high frequency electrical stimulation, the sciatic nerve was harvested one month after injury. Theses nerves were subjected to immunohistochemistry analysis of S-100 and neurofilament (Figure 2A-H). There was significantly higher expression of myelination marker such as S-100 and neurofilament in immediate and late high frequency electrical stimulation groups as compared to control and immediate low frequency electrical stimulation (Figure 2 I, H). This result implicated that high frequency electrical stimulation as either immediate or late administration had the potential for nerve regeneration.<br><br>The synaptophysin and TNF-alpha expression of dorsal root ganglion and somatosensory cortex and hippocampus represented the severity of neuropathic pain. In the immediate high frequency electrical stimulation, there was significantly higher expression of synaptophysin and TNF- over the dorsal root ganglion compared to control or high frequency late electrical stimulation (Figure 3A-H). The higher expression of synaptophysin and TNF- in somatosensory cortex and hippocampus were also noted in the immediate group as compared to late high frequency or control groups (Figure 4 A-N).<br><br>The Catwalk gait analysis demonstrated the motor and sensory functions.. The increased intensity, decreased stance, increases swing, and decreased regularity implicated motor	Page 13-18

		function improvement. However, the increased neuropathic pain produced a reciprocal trend. In the late high frequency electrical stimulation, there were significant improvement of intensity, stance, swing, and regularity index as compared to immediate high frequency electrical stimulation or control groups (Figure 5A-D). This phenomenon showed the effect of immediate high frequency electrical stimulation in motor function compromised by the increased sensory functional impairment.<br>Increased evoked potential in central nervous system showed a positive response to peripheral nervous system injury. The increased evoked potential amplitudes in somatosensory cortex were significantly higher in the immediate high frequency electrical stimulation as compared to late high frequency electrical stimulation or control group (Figure 6A-B). The data of somatosensory evoked potential further confirmed the alteration in neurobehavioral or histomorphologic analysis in the above data.<br><br>To assess the inflammatory response of dorsal root ganglion cells stimulated by the direct electrical stimulation, the dorsal root ganglion cells were harvested and subjected to electrical stimulation. In immunohistochemistry staining, there was documented increased expression of synaptophysin, TNF- and NGF-R in dorsal root ganglion cell culture related to different intensity of electrical stimulation frequency (Figure 7A-R). In the quantitative analysis, there were significant higher expression of synaptophysin, TNF- and NGF-R after high frequency electrical stimulation as compared to low frequency electrical stimulation and control (Figure 7 S, T). Hence, high frequency electrical stimulation harbored the potential to stimulate the dorsal root ganglion cells to express the inflammatory associated proteins.	
Adverse Events	17	There was no adverse events	Page 13-18
DISCUSSION			
Interpretation / Scientific implication	18	The high frequency electrical stimulation harbored the potential to augment nerve regeneration as compared to low frequency electrical stimualtion, but the regeneration ability was compromised by the direct electrical effect on the sensory function impairment. Thus, the appropriate time profile to start the treatment after nerve injury is unclear. In this study, we found that high frequency electrical stimualtion exerted significantly higher effect to prime the dorsal root ganglion cells to express the inflammatory cytokines such as TNF- , synaptophysin, and NGF. In animal study, the significantly increased nerve regeneration was noted in high frequency electrical stimulation either immediate or late, but the immediate electrical stimualtion carries a higher potential to develop neuropathic pain. It seems that a delay in high frequency electrical stimuation should be an appropriate time profile to start the electrical stimualtion after nerve injury.	Page 18-22
Generalisability/Translaction			Page 23

	19 Immediate or late transcutaneous high frequency electrical stimulation had the potential to stimulate the motor nerve regeneration. However, immediate electrical stimulation had a predilection to develop neuropathic pain. It seems that a delay in initiating TENS was a reasonable approach for nerve repair and provided the appropriate time profile in clinical application.	
Funding	20 The authors obtain the funding from the grants of Taichung Veterans General Hospital (TCVGH-1054905C) and conjoint of Taichung Veterans General Hospital/ Providence University (TCVGH-PU1048104).	Page 24

References:
1. Kilkenny C, Browne WJ, Cuthill IC, Emerson M, Altman DG (2010) Improving Bioscience Research Reporting: The ARRIVE Guidelines for Reporting Animal Research. *PLoS Biol* 8(6): e1000412. doi:10.1371/journal.pbio.1000412
2. Schulz KF, Altman DG, Moher D, the CONSORT Group (2010) CONSORT 2010 Statement: updated guidelines for reporting parallel group randomised trials. *BMJ* 340:c332.
